# Raising aluminum foil fists: how to speak about anger in transplant medicine

**DOI:** 10.3389/fsoc.2024.1434500

**Published:** 2025-01-15

**Authors:** Alexandra Vieux Frankel, Eva-Marie Stern

**Affiliations:** ^1^Department of Social Anthropology, York University, Toronto, ON, Canada; ^2^Department of Psychiatry, University of Toronto, Toronto, ON, Canada

**Keywords:** affect, curative imaginaries, transplant, cruel optimism, crip negativity, anger

## Abstract

Dominant narratives of solid-organ transplantation foreground vocabularies of gratitude. Solid-organ transplantation is often celebrated in biomedicine for its high-tech innovation and specialization. But transplantation also includes the organizations that oversee the distribution of donated organs to potential recipients who disproportionately outnumber available organs. Wait-listing for transplant weighs urgency and fitness for transplant against availability, as individuals must simultaneously demonstrate that their conditions are severe enough to warrant transplantation while also showing they are well enough to withstand the transplant procedure that is meant to return the individual from critical illness to able-bodied health. This article considers how promises of cure make affective demands on transplant recipients. Dominant transplantation narratives and metaphors frame transplantation as “rebirth” and the “gift of life.” But this framework constrains transplant recipients' affective and emotional repertoires, positioning gratitude as the primary—if not only—acceptable feeling for performing that the “gift of life” was deserved. Such narrowly sanctioned possibilities for expression elide the affective complexities of transplant recipients' experiences and foreclose opportunities for expressing anger and frustration. This paper unpacks the politics of verbalizing anger among solid-organ transplant recipients at an urban North American hospital. Using arts-based sensory ethnographic interviews with 27 participants, this paper draws on affect theory to understand how transplant recipients critique and protest curative imaginaries while also upholding them. Theorizations from Critical Disability Studies provide generative ways to question negative feelings and more fully understand recipients' experiences.

## Introduction

Anger rarely surfaces in public discourses of solid-organ (heart, kidney, lung, liver, and pancreas) transplantation. Solid-organ transplantation constitutes a highly technical medical arena that intervenes in terminal conditions to extend the lives of transplant recipients. Discourses around transplantation are suffused with positive affective registers that coalesce around hope and gratitude: hope for a return to health and gratitude for the donor's decision, for the donor's kin who upheld the donor's wishes, and for the biomedical practitioners and technologies that make transplantation possible. Transplants are deeply valued by recipients, their loved ones and donor families, for how they extend the lives of recipients—and, through recipients, the lives of donors. However, depictions of solid-organ transplantation as a “miracle” or the “gift of life,” leave little if any space for expressions of affective intensities related to experiences of pre- and post-transplant complications, rejection, and the debilitating effects of the immunosuppressant medications necessary to preventing rejection. Such affective regimes simultaneously foreclose and stigmatize expressions of so-called negative affect.

The promises of transplant medicine to return recipients to a state of health comprise the curative imaginaries in the field. Curative imaginaries situate medical interventions as fixes intended to erase pathology and disability (Clare, 2017; Kafer, 2013). In solid-organ transplantation, the curative imaginaries of biomedicine often fail to account for the complexities of living with transplantation: curative imaginaries depict solid-organ transplantation as a cure to organ failure, creating a neatly bifurcated temporal frame of before and after transplantation (Berkhout et al., 2024). A growing body of social science and humanities literature highlights the ways in which curative imaginaries of biomedicine, with their insistence on medical intervention as fixes that erase pathology and disability (and associate disability with pathology), fail to account for the complexities of living *with* transplantation (Heinemann, 2020; Sharp, 2014). This literature reveals more circular temporalities informed by routine and urgent hospital visits (Heinemann, 2020, 2024), multiple hauntings (McCormack, 2021), and celebrations of technological advancement that fail to take into consideration recipients' often painful embodied experiences (Sharp, 2014). We contribute to this body of literature by asking, What affective demands do curative imaginaries make on solid-organ transplant recipients? And what do expressions of anger reveal about the stakes and politics of transplant medicine's affective registers? These questions have important implications for grappling with the politics of disability as they reveal the pull of curative imaginaries, the desire to protest those imaginaries' affective expectations, and the harms that those imaginaries can produce.

Centering affect directs attention to the intensities and reactions that move through and between bodies—that are atmospheric (Massumi, 2002) and swirling (Stewart, 2007). The term affect has acquired multiple and sometimes conflicting usages and definitions. We draw on Sara Ahmed's and Lauren Berlant's writing on affect as pre-personal feelings that can structure relations, namely Ahmed (2010)'s affect alien and Berlant's cruel optimism (Berlant, 2011). Each concept calls attention to the promises of happy objects and the affective dimensions of the reproduction of social economic structures. We show that curative imaginaries embody relations of cruel optimism (Berlant, 2011), attachments to unrealizable promises, while angry affects in transplant milieus constitute *alien affects*, the dispositions of killjoys (Ahmed, 2010) who do not participate in reproducing affective ecosystems that characterize solid-organ transplantation. Understanding the affective demands that curative imaginaries make on transplant recipients is essential to unmasking the affective expectations of a so-called good life. We turn to examinations of tragedy, pain, and grief in Critical Disability Studies to deconstruct and reconsider how so-called negative experience is produced and conceptualized—to imagine, instead, anger as affirming of life (Abrams and Adkins, 2020).

This article considers how transplant recipients in a small qualitative study express anger and how they reflect on it. Understanding anger in the context of solid-organ transplantation is essential to identifying the ways in which curative imaginaries make affective demands on transplant recipients. That is, anger is instrumental to apprehending unspoken regimes of affective politics in transplant medicine. We found that expressions of anger were verbal and material, emerging during an arts-based sensory ethnographic interview process. Participants were invited to create foil casts of their hands and forearms that spoke to their transplant experiences. When anger surfaced, it often did so as a clenched fist. The gesture of the clenched fist has been associated with labor, feminist, and civil rights movements across the globe since the early 20^th^ century. We understand participants' fists, evocative of anger, as critiques of the compulsory and sanctioned affects of transplant medicine. These clenched fists highlight the failures of curative imaginaries to create space for so-called negative affects. But participants' foil casts did not celebrate negative affect. They are evidence of wrestling with the cruelty (Berlant, 2011) of curative imaginaries in transplant medicine and the politics of rejecting them. As a result, participants in this study did not *crip* their experience, that is, they did not subvert “mainstream representations or practices to reveal able-bodied assumptions and exclusionary effects” (Sandahl, 2003, p. 37). We argue that these foil fists gesture simultaneously toward resisting and reinforcing the affective demands of curative imaginaries, revealing both their pull and their stakes. Participants engage in the work of trying to make space for anger and other alien affects, but they do so while still reproducing the affective regimes that they protest. The result is a story about how participants materially create space to speak about anger in transplant medicine.

The structure of this article retraces the ways in which anger surfaced and materialized in the arts-based sensory ethnographic interviews from which the data emerged. As a result it does not follow the familiar format of background, methods, results, discussion, and conclusion. Elaborated in the Methods section, these interviews asked participants to recall sensory experiences of transplantation and invited each to make an aluminum foil sculpture that they then transformed. We structured the article in a way that reflects the research process in order to better contextualize our data—that is, participants' stories and foil sculptures—within the epistemological and ontological frameworks from which they emerged (Barad, 2007). Configuring our research in this way draws inspiration from feminist anthropology and science and technology studies (STS) literatures that understand knowledge production as profoundly situated (Haraway, 1988; Abu-Lughod, 1991). Feminist approaches to knowledge production often foreground personal stories (verbal and arts-based) and demonstrate how individual experience is entangled in, and informed by, historical and sociopolitical processes (Hartman, 2008; Sharpe, 2016). Centering personal stories importantly counters tendencies toward abstraction and the harmful erasures that abstraction engenders. This work of situating participants' contributions becomes even more important for social science and humanities research in biomedical arenas. Biomedicine's narrow epistemic frame (Squier, 2007) of what forms of information are salient—alongside the tendencies to value abstraction (Kleinman, 1997) and objectification (Jain, 2013) in biomedicine—makes working with contextualized stories rather than objectified datapoints central to how we conduct and communicate this particular research. Our aim is not to produce generalizable assertions about anger in relation to transplant medicine, but to ask what kinds of affective politics anger can reveal. We demonstrate these connections between individual experience and affective relations by interweaving participants' stories with scholarly discussions in affect theory and Critical Disability Studies. In this way, participants feature in the article not as datapoints or research subjects but as theorists of their own experiences.

We first provide a discussion of our methods, the larger project out of which this research emerged, and the contributions of a small-scale qualitative study. The following section elaborates the contexts in which recipients' foil casts materialized, connecting the casts to recipients' transplant experiences and public imagery of fists as symbols of protest and solidarity. We then examine the affective obligations of curative imaginaries. These obligations reveal how the imaginaries become normalized and hegemonic. The last section draws on Critical Disability Studies literature on tragedy (Abrams and Adkins, 2020), pain (Lau, 2020; Patsavas, 2014), and grief (Crosby, 2019) to problematize the association of so-called negativity with negation. With this reframing, we examine the implications of how participants transformed their casts and intervened in the anger associated with the casts' clenched fists. While participants did not outright reject curative imaginaries, they wrestled with how to make space for anger. Clenched foil fists, then, become calls to recognize the limits of curative imaginaries and the experiences they obscure.

## Methods

The research presented in this article is part of a larger project titled Frictions of Futurity and Cure in Transplant Medicine (“Frictions”). The Frictions research team is an interdisciplinary group of researchers and mental health practitioners, including psychiatrists, Critical Disability Studies scholars, medical anthropologists, an art therapist, artists, and medical students. In-person and participant-facing research began in August of 2022 at a large urban North American transplant center. The Frictions project draws on feminist STS, medical anthropology, and queer and crip theory to generate ways of knowing transplant experiences differently. Transplant medicine is often hailed as the height of biomedical achievement. While metaphors of transplantation as offering “miracles,” “the gift of life,” and second chances circulate widely in the field, the team sought to understand health and illness in transplantation afresh by examining and complicating transplant medicine's curative imaginaries: What experiences get obscured amid these celebrations? What imaginations and materializations of living, thriving, and grieving unfold when the norms and expectations of transplant medicine are questioned rather than taken for granted? What futures emerge in their wake (Sharpe, 2016)? Research methods include participant observation in transplant—focused clinical liaison psychiatry rounds, a pre-hab and rehab clinic for lung transplant patients, and an outpatient liver transplant clinic; standard and arts-based interviews with transplant recipients; discourse analysis of transplant manuals provided to transplant patients; and sensory ethnographic methods, including sound walks through the hallways and wings where participants in the study were being treated. The Frictions project also supported research creation projects, such as rewriting a liver transplant manual in poetic form, and artist residencies that prioritized artists with lived experience of transplantation and wait-listing. In addition, the Frictions team developed digital stories and art workshops, and hosted public salons, and pop-up art installations. Through these different streams that each engage unique ways of knowing (e.g., through art, discourse, and embodied experiences), the Frictions project sought to illuminate intertwined logics of cure and futurity and their unintended consequences for transplant recipients, those wait-listed, and their families.

Research participants were recruited through multiple streams: recruitment posters were hung in the waiting areas and elevator lobbies where transplant patients would be likely to see them; the transplant medicine clinical liaison psychiatry team shared information about the research with individuals referred to transplant psychiatry, and only those who expressed interest were approached. We shared information about the study with transplant support groups via their newsletters, and participants also circulated the recruitment posters for the study through their own transplant networks. At the time of writing, 27 transplant recipients were interviewed from across solid-organ transplant clinics: three heart recipients, six kidney recipients, sixteen liver recipients, and two lung recipients. The majority of participants identified as women (16), and the remaining identified as men (11). Two individuals identified as queer or gay and one as asexual. Twenty-three participants identified as white North American, two as Middle Eastern, two as Latin American, one as South Asian, and one as Southeast Asian. Two participants were under 40 (one early 20s, the other mid 30s), two participants were in their 40s, and the remaining participants were over 50. Two participants had multiple kidney transplants, and one was waiting for her second kidney transplant.

The research presented in this article focuses on the results from the second interview in a three-interview protocol. Each interview was developed to elicit different forms of engagement and evidence about transplant experiences. The first was a conventional semi-structured interview that asked participants to recount their transplant experiences with a focus on the psychosocial supports that were most meaningful. The second interview brought together sensory and arts-based research methods. This approach was designed to elicit participants' embodied transplant experiences in a trauma-informed way (see Frankel et al., 2024 for further discussion). The third was also a conventional semi-structured interview that focused on the transplant information manuals that were distributed to patients. This third interview queried participants about these manuals, their experiences of the tone and content of the manuals, and how they used them and what they wished to find in them.

The data presented in this article comes from the second, arts-based sensory ethnographic interview. Here, we invited participants to (1) scribble on a piece of paper with whatever mark-making materials were available, then reflect verbally on what they saw; (2) recall aloud their sensory experiences of transplantation; and (3) embody a gesture that spoke to their transplant experience, and based on this gesture, create an aluminum foil cast of their hand and forearm. Once recipients created the cast, they were invited to transform it—paint it, re-shape it, embellish it with further marks, words, or materials—until the cast felt complete (Frankel et al., 2024). The arts-based component was designed to work with art supplies as well as any mark-making materials (e.g., pens and pencils) participants already had available. Interviews were conducted online and in person according to participant preference. Participants' varying levels of comfort for meeting in person, in addition to travel considerations—many participants lived more than two hours away—necessitated online interviews.

This article focuses specifically on expressions of anger that surfaced in arts-based interviews. Although participants shared numerous knotted, complicated, and ambivalent sensory and affective responses in this interview—joy, resolve, dissatisfaction, broken-heartedness, confusion, gratitude, frustration, to name only a few—we specifically engage with anger here. Anger not only interrupts and challenges the taken-for-granted discourses of gratitude and hope that circulate in transplant medicine's milieus, but also holds a mirror back to those expectations. Understanding anger and what makes anger speakable is instrumental to revealing the affective expectations and demands of curative imaginaries in transplant medicine. Seven participants (25.93 percent of participants at the time of writing) explicitly named anger as part of their experience. When participants expressed anger, it tended to be accompanied by the gesture of a fist. Six participants (seven including the wife of one participant who accompanied her husband to the interviews and participated in the mark-making activities) constructed clenched fists as their gesture, which then became the center of their artwork. Fists were thus the most common symbolic response. Participants' verbal comments often expressed complaints about how cold their recovery room was, the incessant beeping of machines, and announcements over the intercom that interrupted much-desired sleep. Others still spoke about how surprised they were at the extremes of pain they experienced post-surgery, with one saying that if asked within the first two weeks of his lung transplant if he would do it again, he would give a resounding “No!”

This article focuses on three of the participants who named anger as part of their transplant experience and one who described the gesture of the fist as symbolic of strength. We decided to center on these four participants for two reasons: to more closely engage with the multiple textures of their stories and experiences, and because these participants vividly connected their foil fists with protest and unfulfilled promises of curative imaginaries. This small-scale study thereby does not offer a generalizable account of solid-organ transplant experiences, or of why and when anger emerges in these contexts. Instead, we take a feminist ethnographic approach that understands personal stories as political (Abu-Lughod, 1993). We examine how participants both hold onto desires for curative imaginaries to be realized, while simultaneously protesting the hegemony of positive affect in transplant medicine. As a result, this research holds a mirror to the cruel optimism (Berlant, 2011) of curative imaginaries and asks how so-called negative affects might be imagined otherwise.

## Aluminum foil fists

When anger surfaced in interviews, it often materialized in the foil casts as clenched fists. Lisa^1^ propped her phone up on her kitchen table so that the camera showed her pressing aluminum foil around her clenched fist. She looked down and then into the camera at us, and exclaimed, “You know what? As I'm doing this, I'm angry! I'm angry! I'm angry and I have a fist and I'm angry.” Lisa's anger took her by surprise. A middle-aged white woman, her reflection on her sensory experiences of transplantation revealed anxiety-laden hallucinations and slips in and out of consciousness—in which she could hear those around her but could not move or speak. Lisa had received a liver transplant six years before the interview. She punctuated her memories with explanations that all the feelings and intensities associated with transplantation were just as strong now as they were at the time. Her liver disease led to encephalopathy, a condition in which toxins that the liver would otherwise have filtered from the blood caused hallucinations. At night, the clock hands would slow to a halt, inducing panic that she would forever remain with liver failure. On multiple occasions, she saw doctors entering the hospital room to say that a donor liver had been found, that the liver was a match and was hers, only for her husband to have to later explain that those experiences were hallucinations. She said she could not trust what she saw, only what she heard. Although her husband's voice often offered comfort and reassurance, while in one of these in-between states, she also heard him ask her best friend if he ought to start making funeral arrangements. Lisa remembers screaming silently from inside her body.

While Lisa's anger surprised her, Julia, another participant, was already aware of her frustration. Julia, a woman of color in her mid 30s, received her first kidney transplant as a teenager in the early 2000s. In 2016, her doctor told her abruptly that her kidney “was done” and left the room. She made the painful return to dialysis, and in 2022 received her second kidney transplant. In our first interview, her frustrations coalesced around failures of care, the discrimination she faced at work for needing to accommodate dialysis and its intensely tiring effects on her body, doctors with whom she had to plead to get a letter for her work, financial stress, and receiving incomplete information since 2016 about psychosocial supports. She explained that she relied on the coping strategies learned in the children's hospital during her first transplant—the importance of soothing touch, whether petting a dog or holding onto a soft blanket. The comparative lack of attention to her psychic distress as an adult surprised her. But she didn't label these feelings of anger until the second interview, when she looked up from her aluminum foil cast saying, “It was anger, the fist.”

Christina and Anna created their aluminum fists without hesitation. Christina, a white middle-aged woman with one child, began participation in the research roughly eight months following her kidney transplant. Christina experienced numerous complications before and after her kidney transplant. She spent nearly ten years on the waitlist. After six months on the kidney-pancreas waitlist, she received “the call” but the donor organs were not a match. Three years later, in 2017, she had a stroke and had to be removed from the list. Once she returned to dialysis, she developed heart troubles that again temporarily removed her from the waitlist. In 2022, she received a kidney-only transplant. Nine days later, she went into rejection. She noticed the telltale fever and her husband immediately drove her the two-plus hours to the transplant hospital, where the medical team was able to halt rejection and save the kidney. In the time between the transplant operation and our first interview nearly eight months later, she had fallen and broken her ankle. The break became more complicated due to co-morbidities and necessitated a fiberglass cast that, while not a complication of transplantation, prolonged the isolation and distress that her wait-listing inaugurated. The ankle injury, she explained, stopped her from enjoying the transplant, for which she had waited so long. She added that although she worries over her new kidney every day, it is the ankle injury “that has really caused me a lot of emotion, and a lot of being upset and angry and pissed off.”

Anna, however, did not identify her fist with anger but with strength and power. We met Anna in her hospital bed, and with her permission replaced the notebooks and devices on the bedside table with foil, markers, yarn, and pipe cleaners. Soft-spoken throughout the interview, she made a foil fist immediately. Anna, a middle-aged white woman, had a liver transplant only weeks before our first interview. She was working as a healthcare practitioner and at first attributed early signs of liver cirrhosis to the fatigue of working in a hospital as the COVID-19 pandemic gripped the globe. Upon being waitlisted, the transplant program encouraged her to seek out a living liver donor. They suggested that she post her story to a social media group where someone seeking to donate a kidney or part of their liver might find potential recipients. A woman in the same medical field found her, and they were a match. But the donor liver was too big, and Anna's gall bladder had to be removed to make room. She also suffered painful fluid buildup in her abdomen (ascites) that would seep through the stitches from the transplant surgery. While still in the hospital, she noticed that her right foot was not responding to her; imaging revealed a fracture in her spine. Her aluminum foil fist stood for everything she had weathered and survived: on the foil, she wrote a pound sign (#), medical shorthand for a fracture; the words “pain,” “tears,” and “IV” in pink; and in green “fluid buildup,” “feeling weak,” and “being ignored.” The fist bore all that she had endured by virtue of moving through it.

The gesture of the clenched fist carries multiple connotations. It is the beginning of a punch, a hand clenched in rage, and a protest. The clenched fist is a widely recognized gesture of protest and solidarity. One of its earlier appearances occurred in 1917 as a symbol of labor strikes for the Industrial Workers of the World. In 1972, Ms. Magazine published a photo of Dorothy Pitman Hughes and Gloria Steinem with fists raised. Indeed, the clenched fist of the Black Lives Matter movement “root[s] this contemporary moment in the Black Power movement of the late 1960s and 1970s” (Leverette, 2021, p. 4). Ahmed (2017) connects the raised fist to feminist willfulness, “re-signifying the hands of feminism as protesting hands” in contrast to the hand engaged in domestic work (p. 85). The foil exercise's prompt to create a cast of one's forearm and hand invites a necessary consideration of gestures as traces of affect and communication. Gestures “reveal the inscription of social and cultural laws, transforming our individual movements” into accounts of collective experience (Rodriquez, 2012, p. 6). Lisa, Julia, and Christina's casts connect their anger to protest, while Anna's foil cast testifies to all she endured but had not bargained for as part of her transplant. These entanglements of anger, protest, and endurance raise the questions: What is the object of anger? What is being protested, and how? Understanding the affective demands of transplant medicine is essential to grasping the salience of these foil fists. It is these affective demands that make anger difficult to speak, and shape how anger and protest, once surfaced, are circumscribed and dampened.

## Affective obligations of curative imaginaries

**Figure 1:**
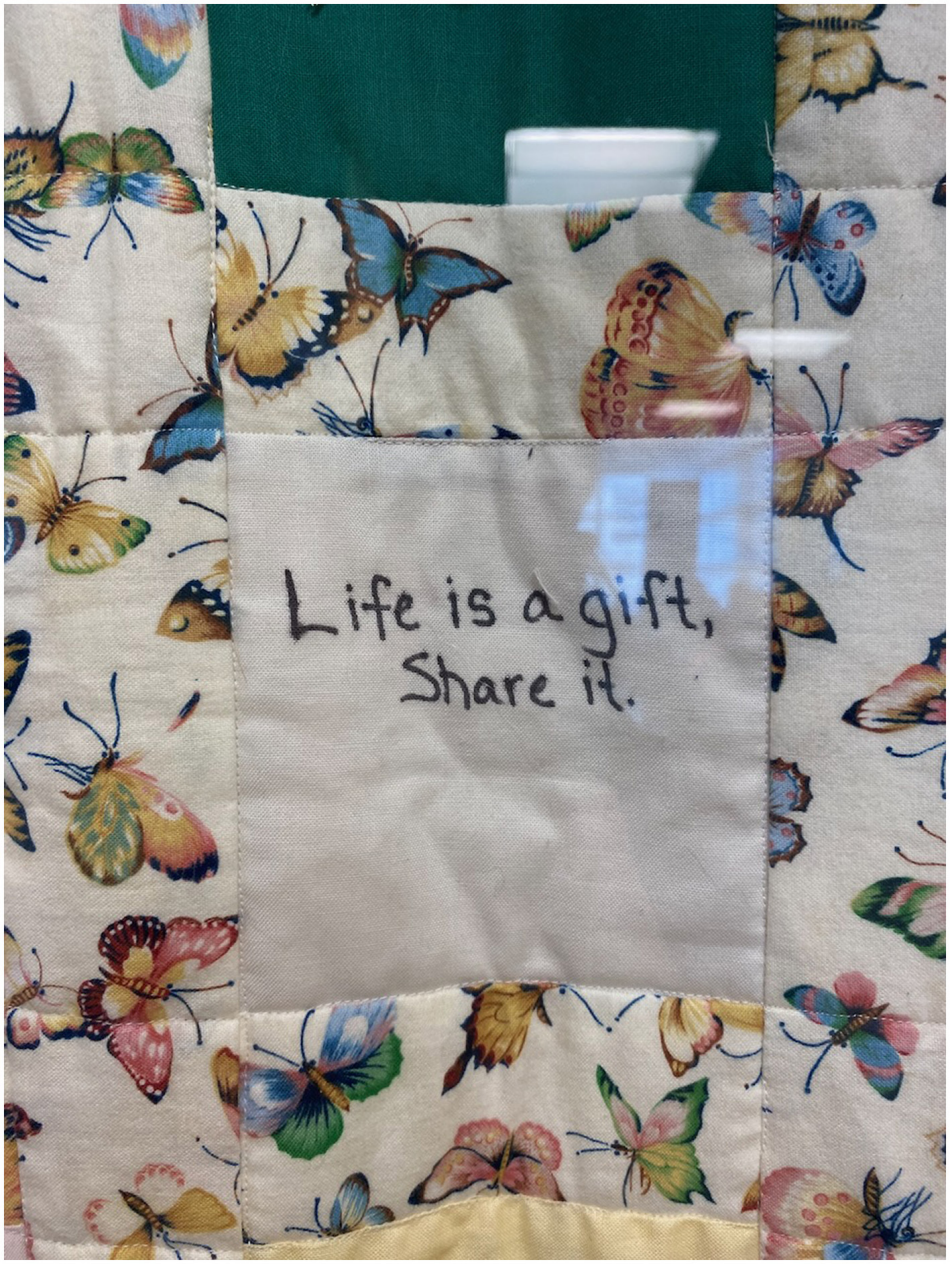
A close up of a quilt that hangs in the waiting room of an outpatient transplant clinic. Donors, recipients, and families have written on squares of fabric in permanent marker. The squares are stitched together with panels with a butterfly pattern. The panel depicted reads, “Life is a gift, share it.” Photo taken by Alexandra Vieux Frankel.

Anger runs against the grain of transplant medicine's dominant affective registers of gratitude and hope. Gratitude functions as a normative and obligatory response to transplantation for recipients. Transplant recipients, donors, donor families, and medical professionals often refer to transplantation as the “gift of life,” making this gift a key metaphor in and outside hospital spaces. References to transplantation as the “gift of life” also adorn clinic walls. In the waiting room of one clinic hangs a quilt whose panels bear notes from transplant recipients, donors, and donor families, offering their thanks, especially to donors and higher powers (Figure 1). Gifts, however, require reciprocation and obligation (Mauss, 2005[1954]), thereby making certain demands on recipients. For Berkhout et al. (2022), these obligations manifest in medical teams' expectations that patients who are wait-listed for transplantation must commit to “full code” status—to being revived via cardiopulmonary resuscitation (CPR), intubation, defibrillation, and medication administration in the event of a medication emergency like cardiac or respiratory arrest. That is, transplant candidates are expected to reject the option to have do-not-resuscitate orders, in order to demonstrate their commitment to the life that transplantation offers, no matter its terms. The affective registers of this commitment to life coalesce around what Shildrick (2015) refers to as the “rhetoric of hope” that “leaves little room for any exploration or understanding of negative affects and emotions that recipients may experience” (p. 21). Heinemann (2020), in her ethnographic work on experiences of solid-organ transplantation in the rural Midwestern United States, similarly describes the hegemonically positive discourse associated with transplantation as a genre unto itself, one that covers over the “more complicated” and “lived” realities of transplantation (p. 1). These emphases on positivity find further connection to transplant technologies (Berkhout et al., 2024). In what Sharp (2014) names “transplant imaginaries,” mainstream praise for the technological advancements that make transplantation and xenotransplantation possible fails to acknowledge the “physical and psychic suffering endured by patients” (p. 3).

Talking about transplantation in registers of hope and gratitude is not only socially sanctioned but obligatory. The salience of gratitude appears in an exchange across several issues of the *American Journal of Transplantation*. Poole et al. (2011) published a small qualitative study that questioned the efficacy of the practice of having transplant recipients author thank-you notes to donor families. Their findings at a Canadian transplant center revealed that recipients struggled to write anonymous thank-you notes to “real people” (any personal or identifying information is redacted by a third party to ensure that the recipient and donor families are kept anonymous) and felt significant distress when notes from donor families were not reciprocated. Poole et al. (2011) conclude that reducing thank-you notes to a technical exercise that limits expression via anonymization is “associated with profound degrees of embodied distress” (p. 621). A letter to the editor, authored by two hepatologists, protesting, stressed that writing thank-you letters was a necessary and cathartic process that relieved rather than induced distress (Selves and Burroughs, 2011). Poole et al. and Selves and Burroughs write from multiple intersections of difference—among them, the former conducting qualitative, multimodal research in Canada, while the latter work in the United Kingdom as practitioners. These different contexts are necessarily also embedded in different power structures, expectations, opportunities, norms, and pressures for narrative (and because Poole et al.'s work is multimodal, also embedded in visual cues). Where and how they collide, however, is most salient here, as they crash in a dispute over the sanctity of gratitude in transplant medicine.

Author and two-time heart transplant recipient Amy Silverstein references similar expectations of gratitude in her *New York Times* guest essay, which was published shortly before her death in 2023. She writes: “Only in transplantation are patients expected to see their disease state as a ‘miracle.' Only in transplant is there pressure to accept what you've been given and not dare express a wish, let alone a demand, for a healthier, longer life” (Silverstein, 2023). The op-ed focuses on stagnation in the development of better immunosuppressive medications for transplant recipients. Required to prevent a transplant recipient's immune system from rejecting the transplanted organ, immunosuppressants also increase vulnerability to bacteria and viruses, increase cancer risk, and can cause kidney damage. Pressure “to see their disease state as a miracle” references the affective demands that curative imaginaries make in transplant medicine.

Anger emerges as a break from affective expectations—as an alien affect (Ahmed, 2010) in solid-organ transplant medicine. Ahmed (2010) develops the concept of “affect aliens” in her discussion of “happy objects” and how the institution of the family “sustains its place as a ‘happy object' by identifying those who do not reproduce its line as the cause of unhappiness” (p. 30). Although Ahmed writes in terms of “affect aliens”—those subjects who refuse to reproduce happy objects—here we draw attention to how affects themselves are made to be alien. “Happy objects” refer to objects of desire. Berlant (2011) discusses happy objects in terms of objects of desire that constitute a “cluster of promises” (p. 23). Happy objects are not necessarily discrete or physical. The happy object, therefore, is not the donor organ itself but the socially associated fantasies of curative imaginaries that are a compact of medical models of cure. That is, the promises of curative imaginaries constitute happy objects, and this affective alignment manifests in normative expressions of hope and gratitude. The vocabularies of desire and promise that Ahmed and Berlant employ are thus especially apt in solid-organ transplantation where curative imaginaries promise a return to health and consequently the erasure of illness and disability (Kafer, 2013; Clare, 2017). While alignment with happy objects yields happy affects, alien affects move in a different direction and thereby contest the sanctity of the happy object. In Ahmed's analysis, queer figures emerge as affect aliens who do not reproduce the imagined norms of the nuclear family. This refusal constitutes a “queer art of failure,” the celebratory failure to be pressured and disciplined into embodying heteronormativity (Halberstam, 2011). Those who express alien affects reject these relations and consequently risk alienation from their objects of desire.

But alignment with the promises of curative imaginaries does not necessarily lead to their realization. Curative imaginaries in transplant medicine can unfold in what Berlant describes as “scenarios of cruel optimism.” Cruel optimism refers to the ways in which attachment to objects of desire also produces distance from those desired outcomes. We may contrast “scenarios of cruel optimism” with “ordinary notions of repair and flourishing” to reveal how our attachment to unrealizable forms of healing can produce harm (Berlant, 2011, p. 49). The tighter one clings to those vaunted scenarios and promises, the more disheartening and painful the outcomes become. Eli Clare describes the yearning for cure as a “connection to loss.” Clare (2017) writes, “What we remember about our body-minds in the past seduces us. We wish. We mourn. We make deals. We desire to return to the days before immobilizing exhaustion or impending death, to the nights 30 years ago when we spun across the dance floor” (p. 57). This form of yearning turns to the past to imagine a future (Clare, 2017), neglecting the ways in which thriving, adapting, and learning unfold in the present.

Project participants often expressed being pulled in multiple directions by grief and yearning. Lisa explained that although her transplant surgery took place more than six years before the interview, she still sometimes feels as though it had happened yesterday—with her fear and anxiety still raw. Anna similarly expressed that her transplant experience unfolded in ways that were wrought with grief. An ultrasound conducted after her surgery brought her to tears. It took three hours for the technician and later the doctor to determine whether blood was indeed moving through the newly transplanted liver. Although she was not explicitly told the reason for the lengthy ultrasound, her experience in healthcare allowed her to piece together what was going on: blood was not moving through the liver and the graft might be lost. Although the ultrasound ultimately found blood flowing and she was discharged from the hospital weeks later, her grief lingered.

In an atmosphere that insists on gratitude and hope as transplant's natural corollaries, how do we understand alien affects such as anger? On the one hand, we may associate so-called negative feelings with complications—that is, so-called negative affects emerge only when curative imaginaries remain unfulfilled. But this narrative acquiesces to the terms of curative imaginaries by reproducing an equivalency between health and “positive” affect. On the other hand, to reject curative imaginaries can risk refusing all medical interventions—interventions that are desired, that have pull, and that can be lifesaving. Yet, as Clare (2017) writes, “the promise of cure can also devalue our present-day selves. It can lead us to dismiss the lessons we've learned, knowledge we've gained, and scars acquired” (p. 61). That is, cure can engender multiple forms of erasure, including of one's own experience.

## Affirming “negative” affect

Normative affective registers sustain transplant medicine's curative imaginaries and fail to make space for negativity—for the recognition of worry, pain, and grief. The social model of disability has been particularly attuned to refuting medical narratives that equate disability with tragedy. The social model shifts attention from individual bodies to the ways in which disability is produced through built environments, providing a necessary correction to medical models that pathologize disability and cast it as needing cure or eradication (Clare, 2017; Siebers, 2008). As a response to the “history of debilitating classifications” endured by bodies with disabilities (Snyder and Mitchell, 2001, p. 374), the social model and its rejection of tragedy results, however, in a lack of attention to lived experience, to phenomenologies of disability. In refusing to engage with tragedy and felt experience, the social model of disability, like the medical model, implicitly likens tragedy to negation and deficiency (Abrams and Adkins, 2020).

Critical Disability Studies' grapplings with negativity can radically redefine tragedy itself. Abrams and Adkins (2020) articulate tragedy as a matter that affirms life rather than negates it. This redefinition of tragedy creates space for dwelling with bodymind pain without reproducing curative imaginaries' harmful associations of disability with tragedy. Abrams and Adkins develop their understanding through an analysis of a Canadian clinic working with families whose children have been diagnosed with Duchenne Muscular Dystrophy. Their term “tragic affirmation” draws on Nietzsche's writing, based in a philosophy of life that relies on neither pessimistic approaches to tragedy nor optimistic ones that avoid discussion of tragedy altogether. Instead, tragedy features as a part of life—not an interruption of it. Abrams and Adkins expand tragic affirmation through their engagement with Spinoza (1994; E4P18S) and Sharp (2011): they build on Spinoza's understanding that bodies cannot be apprehended a priori but must be addressed in context; and on Sharp's attunement to the ways in which power and agency extend beyond human bodies to more-than-human assemblages. As a result, tragic affirmation works against abstract equations that both identify tragedy with disability and “obscure the actual affective relations at work” (Abrams and Adkins, 2020, p. 12).

Tragic affirmation prompts a reconsideration of “negativity” itself. Rather than an attitude that eradicates, removes, or lessens one's vitality, so-called negative affect and experience can instead give rise to sources of life-giving connection. This rearticulation of tragedy builds on reckonings with pain and grief in Critical Disability Studies (although not necessarily in direct conversation) that do the work of articulating the affective relations, atmospheres, and flows entangled with disability. Bodymind pain, while *painful*, is also a source of knowledge and community (Patsavas, 2014; Lau, 2020), and can thereby mitigate the objectifications of ongoing medicalization (Jain, 2013). Patsavas (2014) locates this kind of knowledge in cripistemologies of pain, where cripistemology, a combination of the terms crip and epistemology, refers to “a process of knowledge production that situates pain within discursive systems of power and privilege” (p. 205). Cripistemologies of pain push against the individualization of pain and instead foreground pain as “shared and shareable” (2014, 215). Crosby (2019), similarly, calls for greater attention to experiences of grief in Critical Disability Studies, not as a negation of disability joy, but as part of a refusal to partake in expected narratives “of healing and renewal that end in suffering redeemed” (p. 619). Smilges (2023) describes such feelings in terms of “crip negativity,” which calls attention to “the many bad feelings that disabled, debilitated, and otherwise non-normatively embodyminded people encounter with some regularity: pain, guilt, shame, embarrassment, exhaustion, fear, and anger” (p. 9), while simultaneously critiquing pushes to look toward the future. Indeed, Crosby (2019) draws on Benjamin (1968)'s figure, the Angel of History, who looks backwards at crisis and devastation as a way of moving into the future.^2^ The Angel of History complicates narratives of historical progress, and thereby the belief in cure and technological fixes that propose futures devoid of disability (Kafer, 2013).

These works underscore multiple ways of making room for tragedy, whether in the form of pain or grief. They highlight how tragedy can be rendered as a source of knowledge, a source of connection, and as a way of protesting curative imaginaries, while simultaneously pushing back against the false equivalency between disability and tragedy as negations of life. Such reformulations prompt new observations on the ways in which research participants in this project literally and figuratively handled their anger. That is, research participants engaged in work that embodies the theories we discuss: experimenting with how to make space for anger and how to articulate those experiences and feelings that—while not uncommon—find little expression in “rhetorics of hope” (Shildrick, 2015) and yet may lead to generative connections.

**Figure 2:**
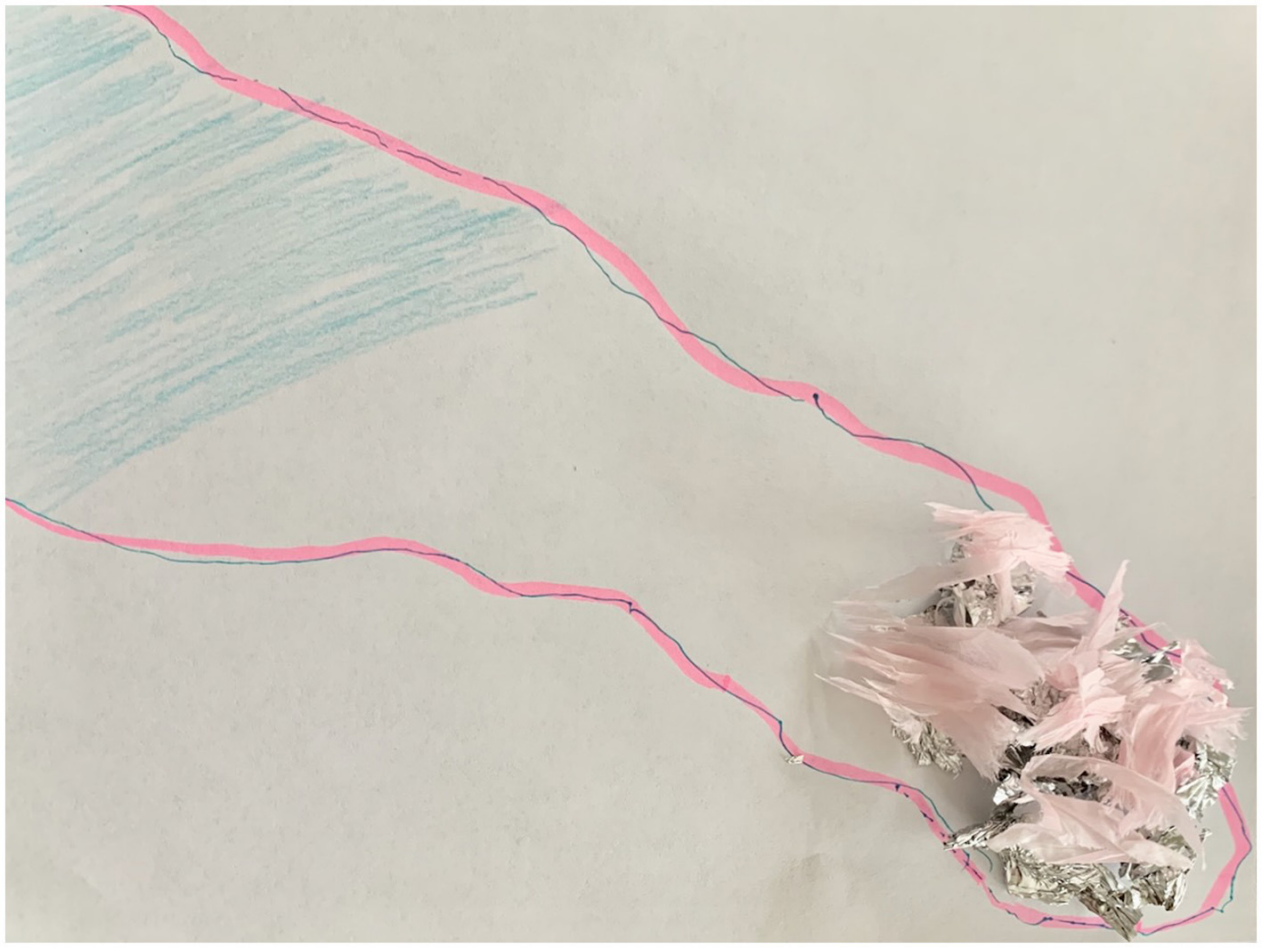
An outline of the participant's forearm and hand drawn in blue pen and pink highlighter. In one corner, near the elbow, the participant filled in the outline with a light blue pencil. At the other end, in the outline of the fist, sits a pile of torn up foil (from the cast) and small pieces of pink tissue paper on top. Photo by participant.

Yet, the space that these participants made for negative affect were carefully partitioned. Lisa, shortly after declaring that she was angry and had made a fist, asked, “Can I break the cast?” Her own forearm had gotten hot in the process of molding the foil around it and her closed fist. After removing the foil from her arm and placing the cast on a blank sheet of paper, she traced the cast's outline in blue and pink, and shaded blue the place on the page that corresponded to where she felt heat (Figure 2). To her, blue was colder and associated with water that she, as a lifelong swimmer, found comforting. She colored the page to change her body's state. She then tore up pieces of pink tissue paper and carefully placed each piece on the fist that she had ripped from the cast forearm. “I only wanted it [my anger] in a spot. I don't want it spilling out anywhere. It has to stay like this pink, it has to stay here…it [the tissue] was softening it, it [the anger] was making it so harsh.” Lisa used the mark-making materials available to ameliorate the anger and heat that she felt—and, more importantly, to isolate the anger and keep it from contaminating the rest of the cast and her transplant experience.

**Figure 3:**
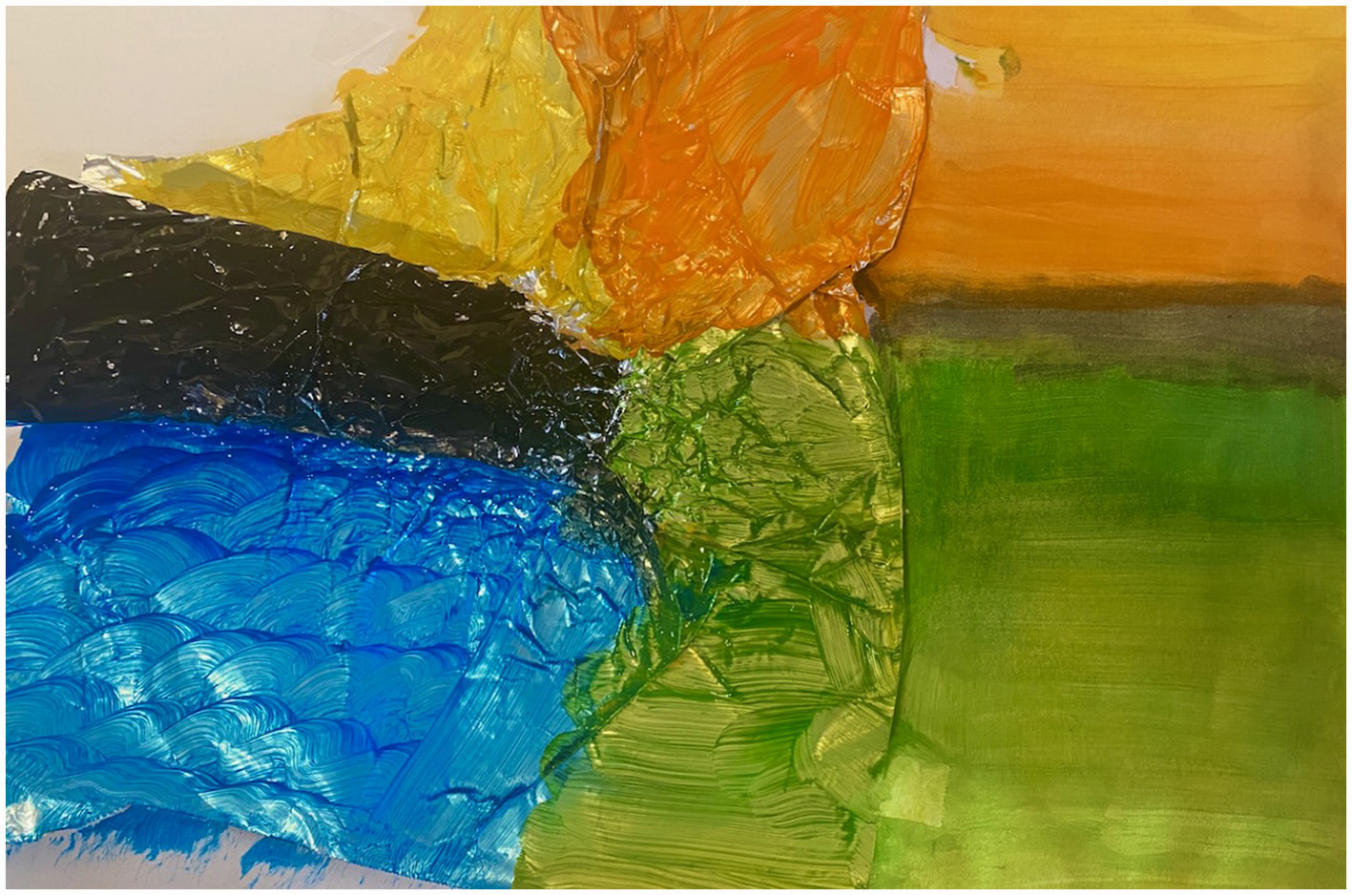
The participant painted the forearm in black, flattened the fist, and surrounded it with bright blue, green, yellow, and orange. These colors spill off of the foil and onto the large sheet of paper beneath it. Photo by Alexandra Vieux Frankel.

Julia similarly used color to intervene in the anger that her clenched fist cast materialized. She placed the foil fist on the left edge of a poster-size sheet of white paper and used tempera paint to cover the entire sheet and foil sculpture. Her forearm was painted black. The color stopped abruptly at her wrist where she started using long green brush strokes. Below her forearm were swirls of blue. And above it, long strokes of yellow and orange with a large block of green to the right (Figure 3). She explained, “I felt like my soul was being drained away.” She continued, “that [is a] fist of anger, and it's like often sometimes that black cloud that can sit over you sometimes with the illness.” This black cloud as a dark space was doubly significant, as she developed a fear of the dark during the hospital stay for her first kidney transplant, a fear that she connects to the uncertainties of falling in and out of comas. The vibrant green, blue, yellow and orange created boundaries around the fist.

Participants used the materials to fence off the angry affects that emerged in the foil exercise. Lisa softened her anger with pink tissue paper. Julia flattened her fist and painted it green. In each of these instances, the materialization of anger was acted upon to ensure that it did not spread. Christina ripped the foil cast in two at the wrist. She crushed one half of the foil into a ball and the other half she carefully flattened, working to smooth it against the table. The crumpled fist, she said, was where she was, and the smoothness where she wanted to be.

In each of these instances, the clenched fist, as anger, was not desirable and it contrasted with desired affects expressed in the softness of pink tissue paper, vibrant colors, and smooth and open qualities. Such contrasts enabled participants to create material fences around their anger. Participants' boundary-making practices—the need to soften and materially contain and separate anger—suggest that they can be rendered as sources of pollution or contamination. Treating anger in this way positions it as *matter out of place*, as something that falls outside established cultural orders and poses a threat to them (Douglas, 2003[1966]; Lugones, 1994)—in this case, a threat to dominant transplant imaginaries and their affective regimes. Boundary-making practices do the important work of creating space for anger in transplant's hegemonically positive affective economies. In so doing they also reaffirm how curative imaginaries position angry affects outside of socially sanctioned affective ecosystems in solid-organ transplantation. Separating matter out of place preserves the purity of social order. That is, the act of cordoning off anger and illness reinforces problematic associations of cure with joy. Participants' transformed clenched fists embody alien affects while simultaneously reproducing the very affective expectations that they protest. Here, curative imaginaries of transplantation are preserved at the same time that they are critiqued. As a result, anger is rendered separate from hope and gratitude.

Participants' initial clenched fists embody gestures of protest. But they also reveal an attachment to objects of desire. The clenched fist “is not merely a symbol of defiance” but also something that, like happy objects, “links subjects to their objects of desire” (Longford, 2020, p. 287). Fists can signal an orientation toward the future, connecting to the hope and gratitude that curative imaginaries sanction. And while a health-giving attitude, hope and gratitude can also become a source of harm when the attitude stigmatizes, erases, and fails to make room for anger and other so-called negative affects. In looking forward with hope, the fist can also signal relations of cruel optimism. Christina's meditations on her transplant experience, after making her foil cast, illustrate the challenges of navigating this charged affective terrain: “My kidney is doing great. I'm thankful for that. That was the main goal.” But she immediately follows it with, “It's this [broken ankle] that has really caused me a lot of emotion, and a lot of being upset and angry and pissed off.” Just as in the foil exercises, anger is distanced from the transplant itself. But her anger around transplantation becomes stickier, more complicated, as she adds, “I worry about my kidney every single day. I'm assuming that's normal, but I haven't had a bit yet where I haven't been able to not worry about it, because as soon as I went into rejection it's been crap.” Christina works to hold gratitude alongside the “crap”: the complications and isolation she endured. Here, she wrestles with how to refuse the affective impositions of transplant medicine's curative imaginaries, while also minimizing (if not eliminating) her alienation from the promises of curative imaginaries. Her broken ankle and graft rejection are named as sources of negative affect—of alien, angry affects. Alternately emphasizing one over the other, we hear her struggle with her loyalty to affective regimes and expectations of curative imaginaries.

The stakes of preserving positive affects are high. Institutions associated with transplantation, whether hospitals, professional associations, or recipient-donor networks, rely on the reproduction of positive affects. Positive affective atmospheres communicate the importance of solid-organ transplantation as a life-saving intervention which purports to eradicate illness. Within solid-organ transplant circles, many worry that expressions of unwelcome outcomes might diminish donor pools and enthusiasm—and thereby undermine the very structures that make transplantation possible (Bartlett, 2023). Participants in our study regularly noted their volunteer work to increase voluntary donation and raise awareness of organ donation, whether through the hospital itself, various organ-specific organizations, or other transplant networks. They are actively engaged in the labor of ensuring that access to transplantation, as imagined through increased donor pools, continues. In this context, finding ways to fence off anger means that participants can express anger—can make room for tragedy, grief, and pain—while still enacting affective regimes that support the enterprise of transplantation.

## Conclusions

How anger is talked about in transplant medicine is inextricably tied to how cure and disability are discussed and imagined. Curative imaginaries in solid-organ transplantation make affective demands on recipients. These imaginaries are wrought with references to transplantation as a “gift of life,” a “miracle,” and a “pinnacle of hope.” Indeed, while transplantation can be a life-saving intervention for many, the affective ecosystem of its imaginaries inhibits acknowledgment of anger and grief, compounding these feelings with shame and embarrassment. As a result, expressions associated with these states come to represent alien affects, those affects that are not aligned with their objects of desire—in this case, promises of cure (Ahmed, 2010). Further, attachments to curative imaginaries can result in scenarios of cruel optimism (Berlant, 2011), where the tighter one clings to promises of a return to health, the greater the distance between onself and the realization of that promise of health. Silverstein (2023) references such relations when she describes organ transplantation as one of the few situations in which individuals are expected to “see their disease state as a miracle.”

It is significant that participants who expressed these alien affects most often did so through the gesture of a clenched fist. Participants started the foil exercise after verbally reflecting on their sensory and embodied transplant experiences, at which time they were invited to choose a gesture that spoke to their experiences and then form an aluminum foil cast around this hand and forearm gesture. Clenched fists emerged again and again. The fists are notable for their associations with protest and solidarity. These aluminum fists embodied demands for recognition of the pain and grief that were part of their transplant experiences but not reflected in the dominant public discourses. As a result, when anger emerged, it first emerged non-verbally, as a fist. But the fists were not all-out rejections of curative imaginaries. Using the foil and other materials present, participants intervened in their anger. They cordoned it off, creating borders around it that would prevent it from seeping into the rest of their foil sculptures. This practice of boundary-making mirrored the interview transcripts, as participants often expressed anger with the caveat that they were grateful for their transplants despite being angry. Boundary-making, thereby, became a way to express alien affects while simultaneously participating in the reproduction of transplant's affective ecosystems.

Neither participants' verbal nor material expressions of anger necessarily embodied tragic affirmation, although their maneuvering to make space for anger does similar work in theorizing how to make anger speakable. Tragic affirmation offers a way to grapple with pain and grief by asserting tragedy as part of life, rather than a negation of it. Indeed, the concept challenges the notion of negativity as negating, showing instead that the negative can also be creative, generative, and cumulative. This is a crip move, a subversive appropriation of tragedy that is turned against the narratives and attitudes that cast disability as tragic and needing eradication or cure (Hamraie and Fritsch, 2019). Tragic affirmation enables anger and other alien affects of transplantation to be understood as life-affirming. *Casting* tragedy as part of life provides important opportunities to explore anger, grief, and pain, while simultaneously acknowledging the harm that curative imaginaries produce. In this way, tragic affirmation invites an exploration of radical ambivalence, the sticky and messy affects involved in seeking medical intervention, while still maintaining a critical eye on curative ideologies, their promises and implications.

## Data Availability

The datasets presented in this article are not readily available because this research generated ethnographic data that includes confidential and highly contextual information. Requests to access the datasets should be directed to frankela@yorku.ca.

## Footnotes

^1^All names are pseudonyms to protect the identities of research participants.

^2^For a discussion of the present in crisis imaginaries, see Wong-Mersereau (2023).
